# Supplementary figures and images for: Stable isotope and fatty acid analyses reveal significant differences in trophic niches of smooth hammerhead Sphyrna zygaena (Carcharhiniformes) among three nursery areas in northern Humboldt Current System
Source: PeerJ. 2021 Apr 22;9:e11283. doi: 10.7717/peerj.11283 (PMC8071072; doi:10.7717/peerj.11283)

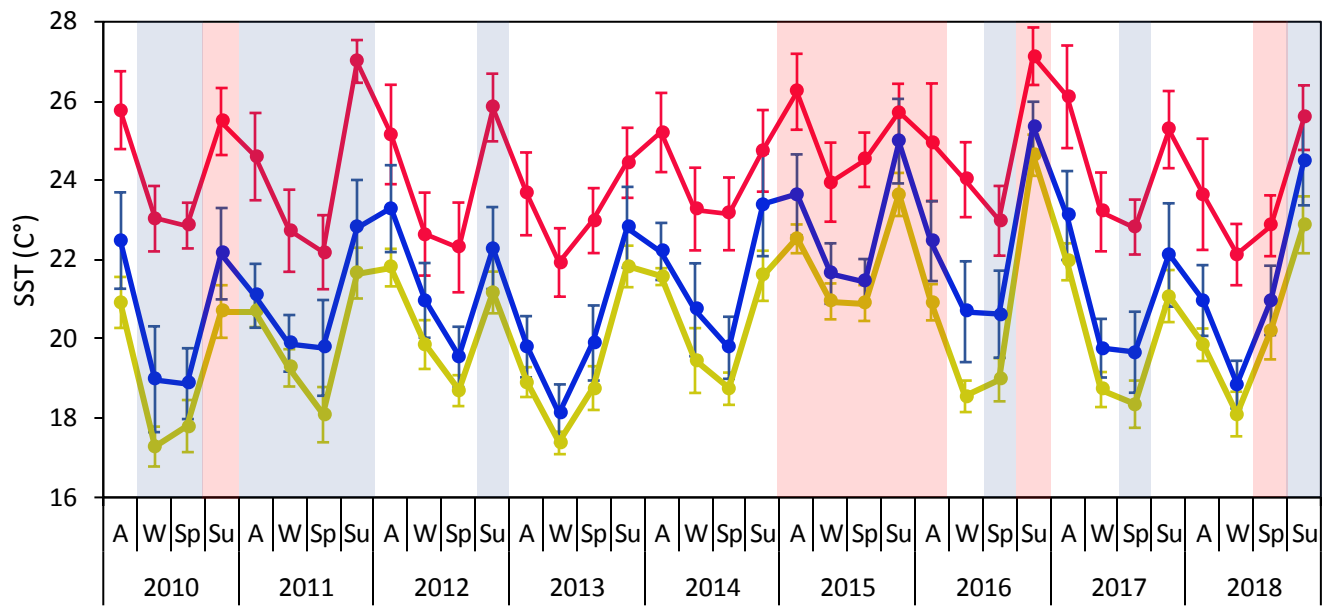

Supplement: Supplemental Information 1 — A, Autumn; W, Winter; Sp, Spring; and Su, Summer. Red background represents El Niño event and blue background represents La Niña event. [file peerj-09-11283-s001.pdf]

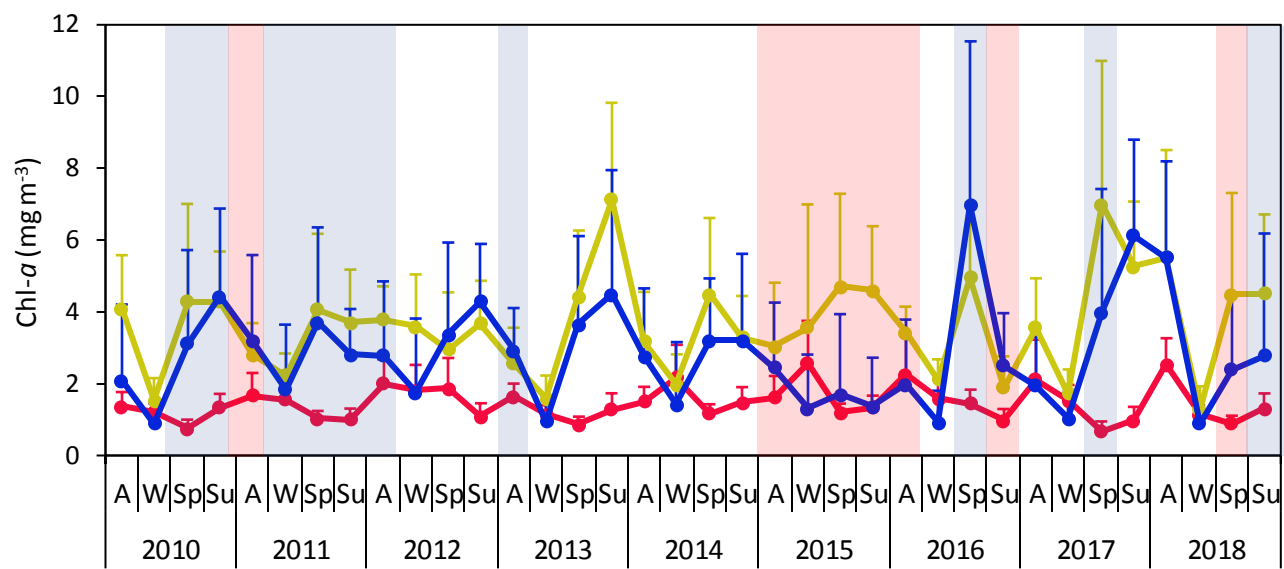

Supplement: Supplemental Information 2 — A: Autumn; W: Winter; Sp: Spring; and Su: Summer. Red background represents El Niño event and blue background represents La Niña event. [file peerj-09-11283-s002.pdf]
