# Supplementary material for: Stable isotope and fatty acid analyses reveal significant differences in trophic niches of smooth hammerhead Sphyrna zygaena (Carcharhiniformes) among three nursery areas in northern Humboldt Current System
Source: PeerJ. 2021 Apr 22;9:e11283. doi: 10.7717/peerj.11283 (PMC8071072; doi:10.7717/peerj.11283)
Supplement: Supplemental Information 3 [file peerj-09-11283-s003.docx]

| **Source** | **D.F.** | **SS** | **MS** | **Pseudo-F** | **P(perm)** | **Unique perms** |
| --- | --- | --- | --- | --- | --- | --- |
| **Area * Tissue** | 5 | 122.16 | 24.431 | 39.7 | 0.001 | 998 |
| **Residuals** | 81 | 49.843 | 0.61535 |  |  |  |
| **Total** | 86 | 172 |  |  |  |  |
