# Supplementary material for: Stable isotope and fatty acid analyses reveal significant differences in trophic niches of smooth hammerhead Sphyrna zygaena (Carcharhiniformes) among three nursery areas in northern Humboldt Current System
Source: PeerJ. 2021 Apr 22;9:e11283. doi: 10.7717/peerj.11283 (PMC8071072; doi:10.7717/peerj.11283)
Supplement: Supplemental Information 4 [file peerj-09-11283-s004.docx]

| **Source** | **D.F.** | **SS** | **MS** | **Pseudo-F** | **P(perm)** | **Unique perms** |
| --- | --- | --- | --- | --- | --- | --- |
| **Area** | 2 | 1060.7 | 530.37 | 4.8135 | 0.004 | 999 |
| **Residuals** | 40 | 4407.3 | 110.18 |  |  |  |
| **Total** | 42 | 5468.1 |  |  |  |  |
